# Meta-Analysis of HTLV-1-Infected Patients Identifies CD40LG and GBP2 as Markers of ATLL and HAM/TSP Clinical Status: Two Genes Beat as One

**DOI:** 10.3389/fgene.2019.01056

**Published:** 2019-11-08

**Authors:** Eduardo Rocha Fukutani, Pablo Ivan Pereira Ramos, José Irahe Kasprzykowski, Lucas Gentil Azevedo, Moreno Magalhães de Souza Rodrigues, João Victor de Oliveira Pimenta Lima, Helton Fábio Santos de Araújo Junior, Kiyoshi Ferreira Fukutani, Artur Trancoso Lopo de Queiroz

**Affiliations:** ^1^Center of Data and Knowledge Integration for Health (CIDACS), Instituto Gonçalo Moniz, FIOCRUZ, Salvador, Brazil; ^2^Laboratório de Análise e Visualização de Dados, FIOCRUZ-RO, Salvador, Brazil; ^3^Fundação José Silveira, Multinational Organization Network Sponsoring Translational and Epidemiological Research, FJS, Salvador, Brazil; ^4^Faculdade de Medicina, Faculdade de Tecnologia e Ciências, Salvador, Brazil

**Keywords:** human T-lymphotropic virus 1, bioinformatics, biomarkers, adult T-cell lymphoma/leukemia, HTLV-1 associated myelopathy/tropical spastic paraparesis, meta-analysis

## Abstract

Human T-lymphotropic virus 1 (HTLV-1) was the first recognized human retrovirus. Infection can lead to two main symptomatologies: adult T-cell lymphoma/leukemia (ATLL) and HTLV-1 associated myelopathy/tropical spastic paraparesis (HAM/TSP). Each manifestation is associated with distinct characteristics, as ATLL presents as a leukemia-like disease, while HAM/TSP presents as severe inflammation in the central nervous system, leading to paraparesis. Previous studies have identified molecules associated with disease development, e.g., the downregulation of Foxp3 in Treg cells was associated with increased risk of HAM/TSP. In addition, elevated levels of CXCL10, CXCL9, and Neopterin in cerebrospinal fluid also present increased risk. However, these molecules were only associated with specific patient groups or viral strains. Furthermore, the majority of studies did not jointly compare all clinical manifestations, and robust analysis entails the inclusion of both ATLL and HAM/TSP. The low numbers of samples also pose difficulties in conducting gene expression analysis to identify specific molecular relationships. To address these limitations and increase the power of manifestation-specific gene associations, meta-analysis was performed using publicly available gene expression data. The application of supervised learning techniques identified alterations in two genes observed to act in tandem as potential biomarkers: *GBP2* was associated with HAM/TSP, and *CD40LG* with ATLL. Together, both molecules demonstrated high sample-classification accuracy (AUC values: 0.88 and 1.0, respectively). Next, other genes with expression correlated to these genes were identified, and we attempted to relate the enriched pathways identified with the characteristic of each clinical manifestation. The present findings contribute to knowledge surrounding viral progression and suggest a potentially powerful new tool for the molecular classification of HTLV-associated diseases.

## Introduction

Human T-lymphotropic virus 1 (HTLV-1) belongs to the *Retroviridae* family and Deltaretrovirus genus, and presents tropism in the infection of T lymphocyte cells (Mirvish et al., 2011). Two diseases are mainly associated with this infection: adult T-cell lymphoma/leukemia (ATLL) and HTLV-associated myelopathy/tropical spastic paraparesis (HAM/TSP) (Gessain and Mahieux, 2012). Around 2–5% of HTLV-infected subjects develop ATLL (Uchiyama et al., 1977) and 0.25–3.8% develop HAM/TSP (Osame et al., 1986), while the majority of HTLV-infected subjects remain asymptomatic (Galvão-Castro et al., 1997). ATLL is a lymphoma-like disease classified into four subtypes: acute, chronic, smoldering, and lymphoma (Shimoyama and members of The Lymphoma Study Group (1984–87)*, 1991). Developing this symptomatology results in a life expectancy less than 1 year in around 65% of affected individuals (Matutes, 2007), in addition to low documented chemotherapeutic response (Yamada et al., 2001). HAM/TSP is characterized as an inflammatory disease of the central nervous system (CNS), can progressively evolve to spastic paraparesis, and results in sensory disturbance in the lower extremities and bladder/bowel dysfunction (Nakagawa et al., 1995).

Currently, ATLL can be diagnosed by integrating cytology and lymphocyte immunophenotyping with HTLV-1 serology (Matutes, 2007). The diagnosis of HAM/TSP is based on clinical evaluation and the exclusion of other disorders and molecular and serological diagnosis, including HTLV-1 serology, Western blotting, and PCR analysis (Yamano and Sato, 2012). In this complex scenario, the identification of biomarkers of this disease is crucial for improving patient care and treatment. With the goal of furthering the understanding surrounding the mechanisms related to disease manifestation, some studies employing gene expression have been conducted. For instance, the downregulation of the FOXP3 gene in T-reg cells was reported to be induced by the HBZ viral protein from HTLV-1. Accordingly, the stimulated proinflammatory response was found to be associated with HAM/TSP development (Yamamoto-Taguchi et al., 2013). Furthermore, other molecules in cerebrospinal fluid, such as CXCL10, CXCL9, and neopterin, have been proposed as promising candidates for prognostic biomarkers of HAM/TSP, offering improved predictive values in comparison to proviral load (Sato et al., 2013).

On the other hand, CAN2 and SPTA2 proteins have been proposed as biomarkers capable of classifying ATLL patients. CAN2 activity was found to induce ATLL cell death and the corresponding gene was downregulated in these cells. In addition, 17 proteins were proposed as capable of classifying healthy controls from asymptomatic carriers (ACs), HAM/TSP, and ATLL patients (Ishihara et al., 2013). Several alterations in anti-inflammatory cytokine levels in infected T cells, e.g., increased IL-10 and suppressed pro-inflammatory cytokines, were also associated with this disease (Kagdi et al., 2018). Another study suggested diagnosing patients by measuring antibody responses to HTLV-1 gag, Env, and Tax proteins (Enose-Akahata et al., 2012); however, this is akin to an immunological diagnosis. Despite the identification of biomarker candidates, various limitations have prevented adoption, as some markers were only identified in specific populations (Yasuma et al., 2016), small sample sizes were used (Ishihara et al., 2013), and the identification was performed only in specific clinical manifestations without appropriate confirmation for use as a general biomarker (Sato et al., 2013; Yamamoto-Taguchi et al., 2013).

To mitigate the impact of low sample sizes, which have limited the interpretation of individual studies, meta-analysis approaches have been employed in the field of gene/marker identification. This approach was used to highlight important genes and molecular pathways in endometrioid endometrial cancer (O’Mara et al., 2016), for the identification of programmed death-ligand 1 as a potential biomarker in glioblastoma (Xue et al., 2017), to identify a set of candidate genes, pathways, and transcription factors not previously associated with the pathogenesis of sickle cell disease (Hounkpe et al., 2015), and to disclose a novel set of candidate genetic markers, pathways, and transcription factors common to both thrombosis and myeloproliferative disorders (Jha et al., 2016). Meta-analysis, in combination with classical approaches and machine learning, has also been applied to identify biomarkers of viral infection in the *Aedes aegypti* mosquito (Fukutani et al., 2017). This methodology has proven powerful in discriminatory classification using gene expression data and was recently highlighted as a potentially useful method for discovering new evidences (Debray et al., 2017); Sweeney et al., 2017). Given the need to identify biomarkers associated with HTLV-1 infection, and considering the abundance of individual studies that resulted in the generation of gene expression datasets, we performed meta-analysis in an attempt to identify candidate transcriptional biomarkers that could offer improved predictive power in the classification of clinical manifestations in HTLV-1, a novelty in this field that has never been done before.

## Methodology

### Description of Datasets Comprising the Discovery Dataset

To identify published datasets relevant to HTLV infection, the Gene Expression Omnibus (GEO) database (https://www.ncbi.nlm.nih.gov/geo/) was searched filtering *Homo sapiens* as the organism of interest and “HTLV” as the keyword. This query returned a total of 41 datasets (search performed in September 2017). After manual evaluation, 32 datasets were excluded due to methodological incompatibility (non-blood cell tissues and absence of symptomatologic information). Of the remaining datasets, three with detailed gene expression by peripheral blood mononuclear cells (PBMCs) were selected to build the Discovery dataset: GSE55851 (Kobayashi et al., 2014), GSE29312, and GSE29332 (Tattermusch et al., 2012). All of the studies that produced these datasets were performed in PBMCs and included at least two different clinical forms of infection, as well as controls (healthy individuals). When combined, the three datasets included 20 controls, 43 AC, 12 ATLL, and 20 HAM/TSP samples (**Table 1**). For our analysis, the AC samples were discarded to avoid possible classification bias, since this form can evolve to another clinical manifestation at some point during the patient’s life, and no information regarding disease progression was provided. The remaining six datasets performed in other tissue types were used for *in silico* validation.

**Table 1:** Description of the datasets used as the Discovery set.

Accession number	Reference	Symptomatology	Sample number	Tissue
GSE55851	Kobayashi et al. (2014)	Control	3	PBMCs
		Asymptomatic	6	PBMCs
		ATLL	12	PBMCs
GSE29312	Tattermusch et al. (2012)	Control	9	PBMCs
		Asymptomatic	20	PBMCs
		HAM/TSP	10	PBMCs
GSE29332	Tattermusch et al. (2012)	Control	8	PBMCs
		Asymptomatic	17	PBMCs
		HAM/TSP	10	PBMCs
Total		Control	20	PBMCs
		Asymptomatic	43	PBMCs
		ATLL	12	PBMCs
		HAM/TSP	20	PBMCs

### Data Retrieval, Pre-Processing, and Batch Correction

Raw expression data were downloaded from GEO/NCBI using the *GEOquery* package (Davis and Meltzer, 2007). Next, the *collapseRows* R function in the *WGCNA* package (Miller et al., 2011) was used to collapse the data, and only probes mapping to genes common to all datasets were maintained. Log transformation was applied to the expression data using the *preProcessCore* package (Bolstad, 2018), and outlier samples were identified and removed by the *ArrayQualityMetrics* package for R (Kauffmann et al., 2008). The *plyr* package was subsequently used to merge all data (Wickham, 2011). Following pre-processing, the combined dataset was submitted to a batch correction procedure using an empirical Bayes framework implemented in the *ComBat* function of the *sva* package (Leek et al., 2013), with clinical manifestations and original datasets as covariates. This allowed us to account for known or unknown sources of variation in the datasets, enabling the use of samples from different datasets in the integrated dataset (i.e., Discovery dataset). This method allowed for the inclusion of the maximum number of samples for analysis, in addition to more robust data interpretation, leading to the identification of consistent insights regarding biological phenomena. *ComBat* has been used in other studies and was shown to outperform other similar tools designed for this purpose (Chen et al., 2011). The final dataset consisted of 94 samples, with expression data pertaining to 10,533 genes in total.

### Classification of HTLV Patient Clinical Manifestation *via* Decision Tree

A decision tree classification procedure was performed in the Discovery dataset to identify the key genes related to HTLV patient clinical manifestation (ATLL or HAM/TSP). Decision trees were constructed using the *rpart* package Therneau et al. (2015), which screens for the key factors that allow for the separation of the groups with maximum accuracy. To measure the performance of the classification model, areas under receiver operating characteristic (ROC) curves were calculated to determine a given model’s sensitivity and specificity. The overall accuracy of a model is calculated by estimating the area under the curve (AUC), permitting measurements of the degree of class separability in a given model. Values approximating 1.0 indicate that the model is suitably capable of distinguishing among different classes. Finally, scatterplots were generated to visualize the dispersion of samples according to the model threshold in order to verify the accuracy estimated by ROC curve analysis.

### Co-Expression and Enrichment Analysis of Genes Related to *CD40LG* and *GBP2*

A correlation matrix between the genes *CD40LG* and *GBP2* (identified as best classifiers) and all the genes within the Discovery dataset was constructed. Correlation was calculated separately for each group (control, ATLL, and HAM/TSP) using gene expression values measured as biweight midcorrelation coefficients, which function similarly to Pearson’s r, except this technique is more robust with regard to data outliers (Langfelder and Horvath, 2012). Correlations were considered significant using a threshold of |r| ≥0.7 and p-value ≤0.05. Next, correlated genes were clustered according to the functional terms of the REACTOME pathway database (https://reactome.org/). This enrichment analysis was performed using *clusterProfiler*Yu et al. (2012) with the following parameters: p-value threshold = 0.05, Q-value threshold = 0.05, minimum number of genes to cluster = 20, maximum number of genes to cluster = 500.

### Description of Datasets Used for Validation

Six microarray expression datasets were retrieved from GEO: GSE17718 (Kress et al., 2010), GSE6034 (Hamamura et al., 2007), GSE38537 (Pinto et al., 2014), GSE33615 (Fujikawa et al., 2016), GSE57259 (Araya et al., 2014), and GSE19080 (no citation available at GEO/NCBI). To confirm the gene signature performance, we performed the gene model comparison in the validation dataset independently, without using the thresholds yielded by the decision tree model estimated during the discovery phase. The model comparison in each different dataset was obtained by applying a logistic regression fitting, which estimated the variable accuracy (CD40LG and GBP2), according to the response variable [determined by dataset metadata (HTLV status)]. Then, the ROC curve and the AUC were measured, which allows the comparison of the gene signature classification power across the validation datasets. A full description of the selected datasets is available in **Table S1**.

## Results

### Gene Expression of *CD40LG* and *GBP2* Permits Accurate Discrimination of ATLL and HAM/TSP Patients

**Figure 1:**
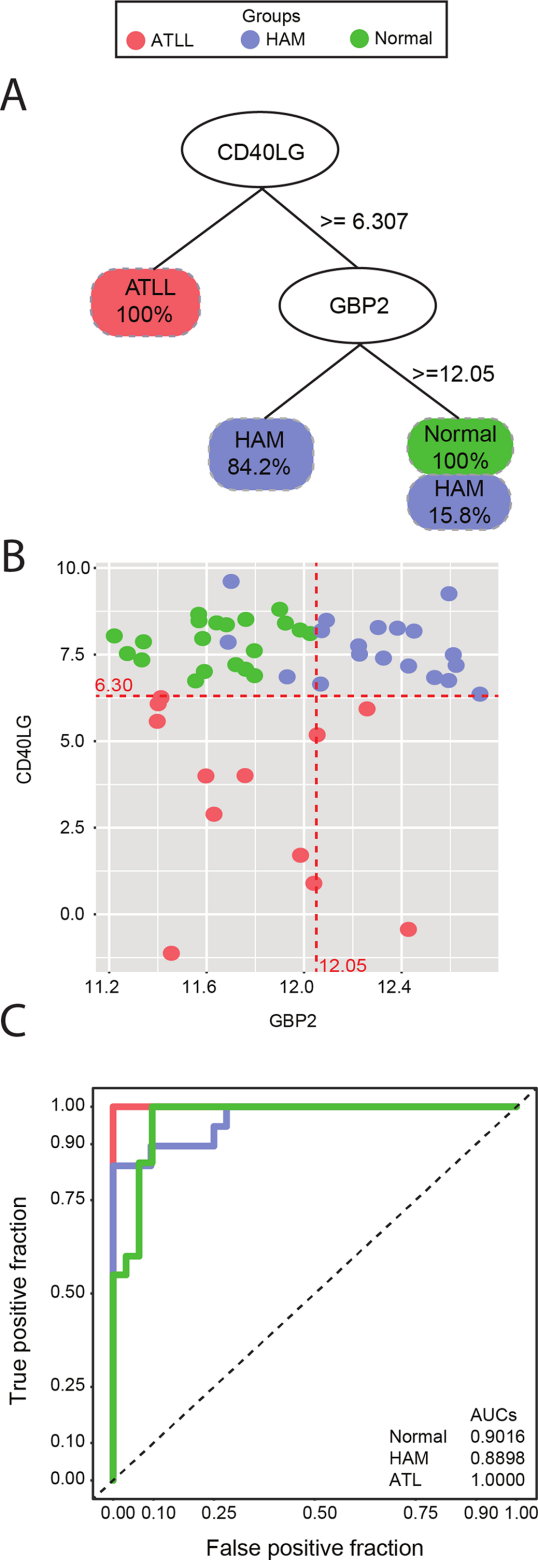
**(A)** Decision tree classification of three different symptomatologies using *CD40LG* to separate all ATLL samples from the others, and *GBP2* to separate 84.2% of the HAM/TSP samples from controls. **(B)** Scatterplot of *CD40LG* (Y axis) and *GBP2* (X axis) gene expression detailing the dispersion of the analyzed samples. Red lines represent the thresholds suggested by decision tree analysis. **(C)** ROC curve representing accuracy. An AUC of 0.9016 was found for the control group, 0.8898 for the HAM/TSP group, and 1.000 for ATLL. The red line represents the ATLL group, blue indicates HAM/TSP, and green is indicative of controls.

The decision tree algorithm identified two genes, *CD40LG* and *GBP2*, as the most informative in differentiating between the clinical manifestations of HTLV-infected samples and controls. The expression of *CD40LG* allowed for the discrimination of individuals with ATLL with 100% accuracy. To correctly classify the remaining samples (HAM/TSP and controls), a second gene (*GBP2*) was required. Expression levels of *GBP2* were able to discriminate HAM/TSP samples with 84.2% classification accuracy, and controls with 100% accuracy, with a 15.8% misclassification rate occurring between HAM/TSP and controls (**Figure 1A**). In addition, sample dispersion was visually checked by scatterplot using the log expression cutoffs returned by the decision tree algorithm: 6.30 for *CD40LG* and 12.05 for *GBP2* (**Figure 1B**). Finally, sensitivity and specificity were measured using ROC curve analysis, revealing high accuracy in discriminating among samples using genes *CD40LG* and *GBP2*: AUC of 0.90 for controls, 0.88 for HAM/TSP, and 1.00 for ATLL (**Figure 1C**).

### Gene Expression of *CD40LG* and *GBP2* Correlate With Various Immune and Metabolic Pathways That Could Impact the Course of HTLV Infection

**Figure 2:**
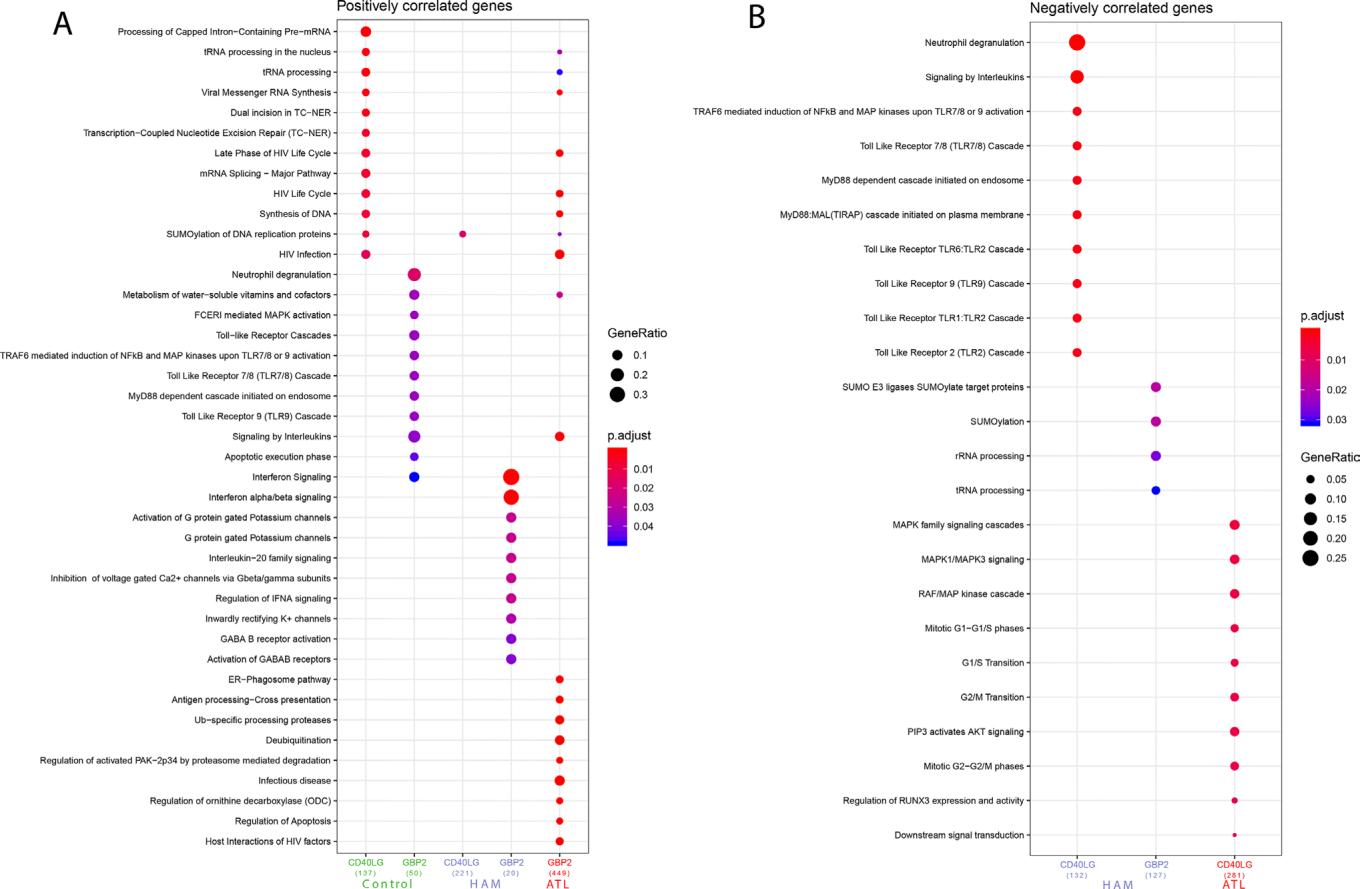
**(A)** Pathways associated with genes found to be positively correlated with CD40LG and GBP2, grouped according to symptomatology. **(B)** Pathways associated with genes found to be negatively correlated with CD40LG and GBP2, grouped according to symptomatology. Analysis performed using the following parameters: p-value = 0.05, q-value = 0.2, minimum number of genes to cluster = 20, maximum number of genes to cluster = 500.

After evaluating the high predictive power of *CD40LG* and *GBP2* in discriminating HTLV clinical status, the roles played by these genes were investigated. Correlation analysis was performed considering global expression for each clinical manifestation (HAM/TSP or ATLL) and controls. Our results showed that 208 genes were significantly positively (r > 0.7 and p-value < 0.05) and 13 genes were significantly negatively (r > 0.7 and p-value < 0.05) correlated with *CD40LG*. Also, 84 genes were significantly positively and 1 gene was significantly negatively correlated with *GBP2*. In contrast, in the ATLL samples, 399 genes were significantly negatively correlated with *CD40LG* and 743 genes were significantly positively correlated with *GBP2*. A total of 12 genes were found to be correlated with both *CD40LG* and *GBP2* (*OAZ1, SLC39A11, NADK, TMED2, SLC38A5, P4HA1, HM13, MGAT2, HIST1H2BG, UQCRFS1, PTDSS1*, and *TAP1B*) (**Figure S1A**). In addition, the HAM/TSP samples presented 394 positive and 420 negative correlations, with three being associated with both *CD40LG* and *GBP2* (*PWP1, H3F3A*, and *GNE*). In these samples, correlations with *CD40LG* were mostly positive, with 367 positive correlations, while those with *GBP2* were mostly negative, with 230 negative correlations (**Figure S1B**). More comprehensive information regarding this correlation analysis and the commonly observed genes is available as supplementary material (**Tables S2**–**S4**). The gene set previously identified correlated with the biomarkers (*CD40LG* and *GBP2*) was analyzed in order to identify their enriched pathways. Thus, the top four pathways identified from being negatively correlated with the *CD40LG* gene set in the HAM/TSP were “Neutrophil degranulation,” “Signaling by interleukins,” “TRAF6-mediated induction of NFkB and MAP kinases upon TLR7/8 or 9 activation,” and “Toll Like Receptor 7/8 (TLR7/8) Cascade.” The main pathways identified from the gene set that negatively correlated with GBP2 in the HAM/TSP were “SUMO E3 ligases SUMOylate target proteins,” “SUMOylation,” “rRNA processing,” and “tRNA processing” (**Figure 2B**). Only one pathway was identified from the gene set that positively correlated with *CD40LG* in HAM/TSP: “SUMOylation of DNA replication proteins.” Several pathways were identified from the genes that were positively correlated with *GBP2* in HAM/TSP: “Interferon Signaling,” “Interferon alpha/beta signaling,” “Activation of G protein gated Potassium channels,” “G protein gated Potassium channels,” and “Interleukin-20 family signaling” (**Figure 2A**).

The top 5 pathways identified from the gene set that negatively correlated with *CD40LG* in the ATLL were “MAPK family signaling cascades,” “MAPK1/MAPK3 signaling,” “RAF/MAP kinase cascade,” “Mitotic G1−G1/S phases,” and “G1/S Transition” (**Figure 2B**). Moreover, the associated pathways from the gene set that positively correlated with *GBP2* in ATLL patients were “tRNA processing in the nucleus,” “tRNA processing,” “Viral Messenger RNA synthesis,” “Late Phase of HIV Life Cycle,” and “HIV Life Cycle” (**Figure 2A**).

By contrast, in the control group, the pathways identified from the gene set that correlated with *CD40LG* were “Processing of Capped Intron-Containing Pre-mRNA,” “tRNA processing in the nucleus,” “tRNA processing,” “Viral Messenger RNA Synthesis,” “Dual incision in TC-NER,” “Transcription-Coupled Nucleotide Excision Repair (TC-NER),” “Late Phase of HIV Life Cycle,” “mRNA Splicing—Major Pathway,” “HIV Life Cycle,” “Synthesis of DNA,” “SUMOylation of DNA replication proteins,” and “HIV infection.” With regard to GBP2’s positively correlated genes, the following pathways were found in the control group: “Neutrophil degranulation,” “Metabolism of water-soluble vitamins and cofactors,” “FCERI mediated MAPK activation,” “Toll-Like Receptors Cascades,” “TRAF6 mediated induction of NFkB and MAP kinases upon TLR7/8 or 9 activation,” “Toll Like Receptor 7/8 (TLR7/8) Cascade,” “MyD88 dependent cascade initiated on endosome,” “Toll Like Receptor 9 (TLR9) Cascade,” “Signaling by Interleukins,” “Apoptotic execution phase,” and “Interferon signaling” (**Figure 2A**). Further information regarding the pathways associated with these genes (ENTREZ ID) is available as supplementary material, separated into negatively correlated (**Table S5**) and positively correlated categories (**Table S6**).

### Validation of *CD40LG* and *GBP2* in Independent Datasets Reveals Classification Robustness in Different Tissue Types

To validate the accuracy of our two-gene model in the discrimination of ATLL, HAM/TSP, and control samples, this model was applied to the other datasets not used in the discovery set: (Kress et al., 2010) (GSE17718), (Hamamura et al., 2007) (GSE6034), (Pinto et al., 2014) (GSE38537), (Yamagishi et al., 2012) (GSE33615), (Olière et al., 2010) (GSE57259), and GSE19080. After downloading and pre-processing these datasets, ROC curve analysis was applied to measure the discriminant power of *CD40LG* and *GBP2* in classifying HLTV-1 clinical manifestations. The discriminant power of this two-gene signature was found to be very high, allowing for the discrimination of the HTLV-1 clinical status in five of the datasets with an AUC value of 1 (GSE17718, GSE6034, GSE38537, GSE33615, and GSE57259). The need to include both genes for accurate classification was evidenced in the GSE19080 dataset (in which the *CD40LG* gene is absent), yielding a much lower AUC (0.875) in the discrimination of control samples, compared to 0.666 for HAM/TSP samples and 0.5 when discriminating ATLL samples. These validation datasets were derived from a variety of tissues, such as cell lines (StEd, MT-2, Tay and MT-4), CD4 lymphocytes, and PBMCs. The overall accuracy of this two-gene signature model is delineated in **Table 2**. Also, the sample distribution using the two-gene expression in all validation dataset is summarized in **Figure S2**.

**Table 2:** Performance of the two-gene signature classifying the samples from validation datasets.

Accession number	Symptomatology	Tissue	Biomarkers	AUC
GSE17718	Control	CD4+ Lymphocyte	CD40LG and GBP2	1.00
	ATLL	Cell lines StEd and MT-2	CD40LG and GBP2	1.00
GSE6034	Control	CD4+ Lymphocyte	CD40LG and GBP2	1.00
	ATLL	Cell lines TaY, MT-2 and MT-4	CD40LG and GBP2	1.00
GSE38537	Control	CD4+ Lymphocyte	CD40LG and GBP2	1.00
	HAM/TSP	CD4+ Lymphocyte	CD40LG and GBP2	1.00
GSE33615	Control	CD4+ Lymphocyte	CD40LG and GBP2	1.00
	ATLL	PBMCs (Mostly CD4+ Lymphocytes)	CD40LG and GBP2	1.00
GSE19080	Control	CD4+ Lymphocyte	GBP2	0.87
	ATLL	CD4+ Lymphocyte	GBP2	0.50
	HAM/TSP	CD4+ Lymphocyte	GBP2	0.66
GSE57259	Control	CD4+ CD25+ CCR4+ Lymphocytes	CD40LG and GBP2	1.00
	HAM/TSP	CD4+ CD25+ CCR4+ Lymphocytes	CD40LG and GBP2	1.00
	ATLL	CD4+ CD25+ CCR4+ Lymphocytes	CD40LG and GBP2	1.00

## Discussion

To date, few studies have attempted to identify biomarkers capable of discriminating between ATLL and HAM/TSP in HTLV-1 infection. A previous report (Sato et al., 2013) suggested three potential prognostic biomarkers in cerebrospinal fluid for HAM/TSP disease progression: CXCL10, CXCL9, and neopterin. Another study (Baratella et al., 2017) stated that the HBZ protein, exclusively localized in the cytoplasm, could be a biomarker of HAM/TSP. In addition, CAN-2 and SPTA-2 were identified as biomarkers capable of discriminating ATLL (Ishihara et al., 2013). However, these biomarkers were found in a specific population and, to the best of our knowledge, the literature contains no sets of biomarkers offering sufficient accuracy to reliably identify both the ATLL and HAM/TSP phenotypes. With the objective of achieving accurate discrimination, we employed a robust bioinformatic approach to consolidate the available expression data using three different datasets combined into a single Discovery dataset. Three studies were selected for this analysis, one submitted by Kobayashi et al. (acc number: GSE55851) and two submitted by Tattermusch et al. (acc number: GSE29332 and GSE29312). The study by Kobayashi et al. compares gene expression levels in PBMCs from ATLL, asymptomatic, and control patients. The other studies submitted by Tattermusch et al. compared gene expression levels in PBMCs from HAM/TSP, asymptomatic, and control individuals. Next, a data mining technique was applied to the merged, batch-corrected Discovery dataset to identify which variables (genes) could effectively discriminate clinical status among the samples. Decision tree analysis revealed genes *CD40LG* and *GBP2* as discriminators of ATLL and HAM/TSP, offering accuracy rates of 100% and 84.2%, respectively. A previous report identified lower *CD40LG* expression in cells expressing PTHrP and MIP-1α, two proteins associated with ATLL progression (Shu et al., 2012). The second marker identified herein, *GBP2*, was previously associated with tax protein activity in HTLV-1 (Arainga et al., 2012). Despite identifying these associations, no previous studies proposed either of these genes as biomarkers of ATLL or HAM/TSP symptomatology.

The *CD40LG* gene encodes a protein located on the surface of T cells and exerts the role of regulating B cell functions (Stelzer et al., 2016). *GBP2* is a guanylate binding protein induced by IFN-γ and is considered as a control factor for tumor cell proliferation and spreading (Messmer-Blust et al., 2010). Our functional approach entailed the correlation of these biomarkers with the global expression of other genes, followed by enrichment analysis using the REACTOME database (Fabregat et al., 2018). This analysis showed that the genes positively correlated with *CD40LG* are associated with pathways mainly related to tRNA processing, viral replication, and mRNA splicing in the control group. However, in the HAM/TSP group, these genes were only found to be associated with the SUMOylation of DNA replication pathway, which is specifically associated with transcription and replication pathways. In addition, the genes negatively correlated with *CD40LG* were found to be associated primarily with neutrophil degranulation, signaling for interleukins and several cascades of Toll Like Receptors in HAM/TSP patients. These pathways may be associated with immune responses involving inflammation (Faurschou and Borregaard, 2003; Lacagnina et al., 2018; Weitzman, 2003), which is frequently observed in HAM/TSP patients (Nakagawa et al., 1995).

On the other hand, the genes negatively correlated with *CD40LG* were found to be associated with MAPK cascade-associated pathways and cell cycle-related pathways. MAPK cascade-related pathways are associated with a wide spectrum of metabolic pathways related to cell proliferation, differentiation, and apoptosis (Shaul and Seger, 2007). Cell cycle-related pathways, such as Mitotic G1-G1/S phases, G1/S Transition, G2/M Transition, and Mitotic G2-G2/M phases, are related to cell proliferation (Matson and Cook, 2017). These pathways are all related to cell proliferation, which is consistent with ATLL symptomatology and the uncontrolled proliferation of T cells (Shimoyama and members of The Lymphoma Study Group (1984–87)*, 1991).

The top pathways that positively correlated with *GBP2* were mainly related to HIV infection, tRNA, and viral mRNA processing and synthesis, signaling by interleukins, and apoptosis regulation. The pathways observed to be related to HIV infection may be due to similarities between HTLV-1 and HIV, as both these retroviruses mainly infect T CD4+ lymphocytes. The tRNA and viral mRNA pathways are associated with the highly active processing of RNAs that occurs in ATLL cells. Furthermore, the regulation of apoptosis could be associated with the immortalization of T CD4+ cells that characterizes the leukemic aspect of ATLL (Bellon et al., 2010).

In order to evaluate the predictive power of the *CD40LG/GBP2* two-gene signature in the accurate classification of HAM/TSP and ATLL samples, we conducted a validation step using independent datasets, which revealed excellent predictive values. The majority of datasets returned an AUC of 1.0, corresponding to an accuracy rate of 100% when classifying samples as ATLL, HAM/TSP, or controls. In one of six validation datasets (GSE19080), a poorer classification accuracy was found, which is likely due to the absence of the *CD40LG* in the array, indicating the requirement of both genes in order to maintain reliably consistent classification. Additionally, the selected validation datasets sampled not only PBMCs but also several transformed cell lines, including MT-2, MT-4, StEd, and TaY, as well as isolated CD4+ cells. These high rates of accuracy seen in a diverse range of tissue types serve to confirm the robustness of the two-gene signature identified herein, suggesting a conserved mechanism in the regulation of genes associated with each symptomatology. Despite some limitations such as the absence of available datasets studying HTLV-1 biomarkers in a transcriptional approach and the reduced sample numbers, our findings provide useful biomarkers to independently identify populations affected by HTLV-1.

## Conclusion

Our meta-analysis of gene expression datasets in HTLV-1-infected patients with specific disease manifestations identified a two-gene signature (*CD40LG/GBP2*) allowing for excellent classification of the HAM/TSP and ATLL phenotypes. This signature was subsequently validated in six independent datasets. An exploratory functional enrichment analysis of the genes found to be positively and negatively correlated with this signature revealed diverse activation and repression of pathways relevant to this viral disease. Our findings add to the accumulation of knowledge surrounding HTLV-1 infection and may contribute to early diagnosis, as well as the treatment of related symptomatologies.

## Data Availability Statement

Publicly available datasets were analyzed in this study. This data can be found here: GSE55851, GSE29312,GSE29332,GSE17718,GSE6034,GSE38537,GSE33615,GSE57259,GSE19080.

## Author Contributions

EF, AQ, KF, MR and PR participated in the data analysis. EF, AQ, KF and PR participated in the manuscript writing. JK, LA, JL and HJ participated in the idea generation for this work.

## Funding

AQ acknowledges financial support from the program Inova Fiocruz (Project number VPPIS-001-FIO18).

## Conflict of Interest

The authors declare that the research was conducted in the absence of any commercial or financial relationships that could be construed as a potential conflict of interest.

## Abbreviations

ATLL, Adult T-Cell Lymphoma/Leukemia; HAM/TSP, HTLV-associated myelopathy/tropical spastic paraparesis; AC, Asymptomatic Carriers.

## Supplementary Material

Table S1 Datasets used on the validation step's detailed information

Table S2 Correlations observed between CD40LG and GBP2 in the HAM/TSP group.

Table S3 Correlations observed between CD40LG and GBP2 in the ATLL group.

Table S4 Correlations observed between CD40LG and GBP2 in the control group.

Table S5 Detailed information regarding the negatively correlated pathways and associated genes (ENTREZ ID).

Table S6 Detailed information regarding the positively correlated pathways and associated genes (ENTREZ ID).

Figure S1 Correlation network based on gene expression values in ATLL samples. Highlighted genes were found to correlate with both CD40LG and GBP2. B - Correlation network based on the gene expression values in the HAM/TSP group. Highlighted genes were found to correlate with both CD40LG and GBP2. C - Correlation network based on the gene expression values in the control group.

Figure S2 Scatterplot of validation datasets sample distribution using the CD40LG and GBP2's log transformed expression values. The samples can be separated by symptomatology [ATLL (green), HAM/TSP (red) and control (blue)], this separation is shown by the collored ellipses. The GSE19080's scatterplot has only GBP2 within the dataset, the values of X and Y axis are both representing GBP2's log transformed expression value.
