# Supplementary figures and images for: Meta-Analysis of HTLV-1-Infected Patients Identifies CD40LG and GBP2 as Markers of ATLL and HAM/TSP Clinical Status: Two Genes Beat as One
Source: Front Genet. 2019 Nov 8;10:1056. doi: 10.3389/fgene.2019.01056 (PMC6857459; doi:10.3389/fgene.2019.01056)

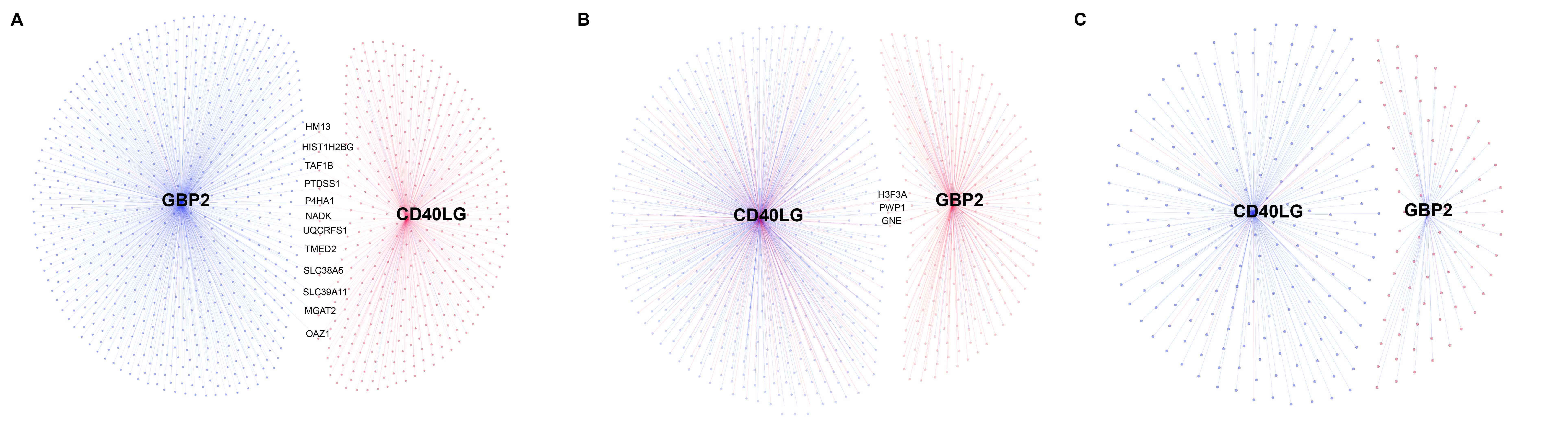

Supplement: Figure S1 — Correlation network based on gene expression values in ATLL samples. Highlighted genes were found to correlate with both CD40LG and GBP2. B - Correlation network based on the gene expression values in the HAM/TSP group. Highlighted genes were found to correlate with both CD40LG and GBP2. C - Correlation network based on the gene expression values in the control group. [file Image_1.jpeg]

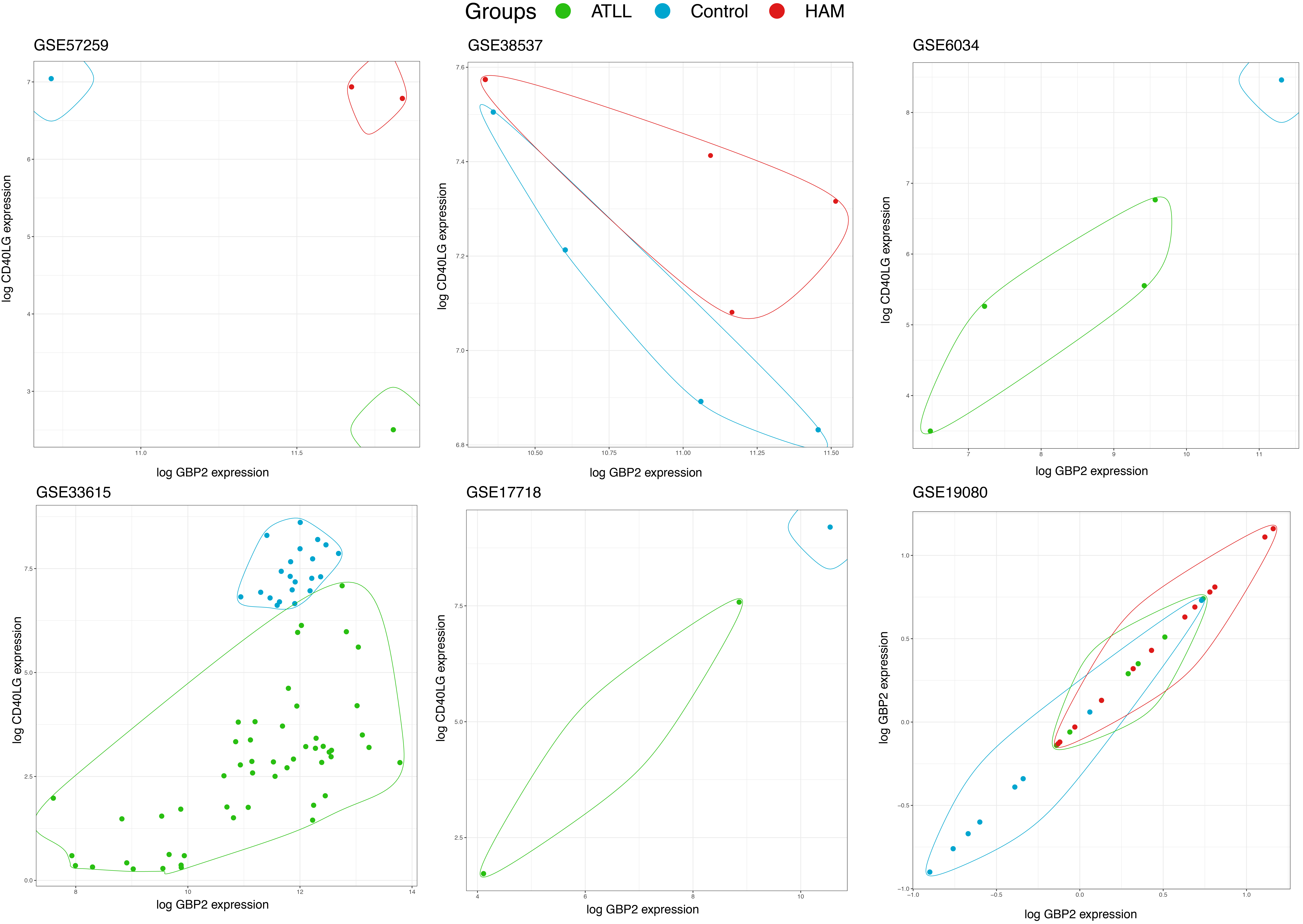

Supplement: Figure S2 — Scatterplot of validation datasets sample distribution using the CD40LG and GBP2's log transformed expression values. The samples can be separated by symptomatology [ATLL (green), HAM/TSP (red) and control (blue)], this separation is shown by the collored ellipses. The GSE19080's scatterplot has only GBP2 within the dataset, the values of X and Y axis are both representing GBP2's log transformed expression value. [file Image_2.png]
